# Functional Characterization and Structure-Guided Mutational Analysis of the Transsulfuration Enzyme Cystathionine γ-Lyase from *Toxoplasma gondii*

**DOI:** 10.3390/ijms19072111

**Published:** 2018-07-20

**Authors:** Elena Maresi, Giacomo Janson, Silvia Fruncillo, Alessandro Paiardini, Rosario Vallone, Paola Dominici, Alessandra Astegno

**Affiliations:** 1 Department of Biotechnology, University of Verona, Strada Le Grazie 15, 37134 Verona, Italy; elena.maresi@univr.it (E.M.); silvia.fruncillo@univr.it (S.F.); rosario.vallone@univr.it (R.V.); alessandra.astegno@univr.it (A.A.); 2 Department of Biochemical Sciences “A. Rossi Fanelli”, Sapienza University of Rome, P.le Aldo Moro 5, 00185 Rome, Italy; giacomo.janson@uniroma1.it (G.J.); alessandro.paiardini@uniroma1.it (A.P.)

**Keywords:** *Toxoplasma gondii*, cystathionine γ-lyase, pyridoxal-5′-phosphate, reverse transsulfuration pathway, reaction specificity

## Abstract

Sulfur-containing amino acids play essential roles in many organisms. The protozoan parasite *Toxoplasma gondii* includes the genes for cystathionine β-synthase and cystathionine γ-lyase (TgCGL), as well as for cysteine synthase, which are crucial enzymes of the transsulfuration and de novo pathways for cysteine biosynthesis, respectively. These enzymes are specifically expressed in the oocyst stage of *T. gondii*. However, their functionality has not been investigated. Herein, we expressed and characterized the putative CGL from *T. gondii*. Recombinant TgCGL almost exclusively catalyses the α,γ-hydrolysis of l-cystathionine to form l-cysteine and displays marginal reactivity toward l-cysteine. Structure-guided homology modelling revealed two striking amino acid differences between the human and parasite CGL active-sites (Glu59 and Ser340 in human to Ser77 and Asn360 in toxoplasma). Mutation of Asn360 to Ser demonstrated the importance of this residue in modulating the specificity for the catalysis of α,β- versus α,γ-elimination of l-cystathionine. Replacement of Ser77 by Glu completely abolished activity towards l-cystathionine. Our results suggest that CGL is an important functional enzyme in *T. gondii*, likely implying that the reverse transsulfuration pathway is operative in the parasite; we also probed the roles of active-site architecture and substrate binding conformations as determinants of reaction specificity in transsulfuration enzymes.

## 1. Introduction

The sulfur-containing amino acids cysteine and methionine play indispensable roles in a wide range of cellular processes and activities, such as synthesis, stability, structure, regulation of catalytic function, and posttranslational modification of many proteins.

Despite the biological importance of these amino acids and their metabolic intermediates in virtually all organisms, genome-wide and functional analyses of the enzymes implicated in their metabolic pathways have revealed notable heterogeneity among protozoan parasites and between parasites and their mammalian hosts.

**Figure 1:**
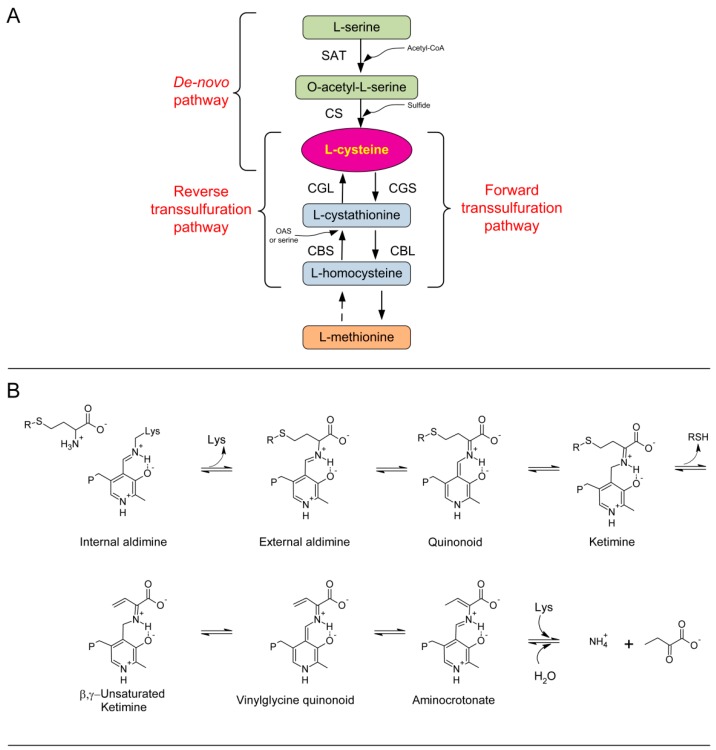
Overview of cysteine metabolism and of reaction catalyzed by CGL. (**A**) Cysteine production via a de novo pathway involving serine acetyltransferase (SAT) and cysteine synthase (CS) from serine in the presence of reduced sulfur. Forward transsulfuration pathway involving cystathionine γ-synthase (CGS) and cystathionine β-lyase (CBL) using the substrate l-cysteine to produce l-homocysteine via the intermediate l-cystathionine. Reverse transsulfuration pathway including cystathionine β-synthase (CBS) and cystathionine γ-lyase (CGL) using the substrate homocysteine in the presence of serine or OAS with an intermediate cystathionine. Dashed arrow implicates several enzymatic reaction steps which were omitted for clarity. (**B**) Mechanism of the γ-elimination reaction in CGL. The substrate is cystathionine and R = CH_2_CH(NH_3_^+^)COO^−^.

In mammals, synthesis of cysteine occurs via the so-called reverse transsulfuration pathway, which involves two pyridoxal-5′-phosphate (PLP)-dependent enzymes, cystathionine β-synthase (CBS, EC 4.2.1.22) and cystathionine γ-lyase (CGL, EC 4.4.1.1). CBS produces cystathionine from homocysteine and serine via a β-replacement reaction, while CGL hydrolyzes cystathionine via an α,γ-elimination reaction to yield cysteine, α-ketobutyrate, and ammonia [1] (Figure 1A).

In contrast to animals, a transsulfuration pathway in the reverse direction (forward transsulfuration) is operative in plants, bacteria, and fungi, in which methionine is synthesized from cysteine via the two complementary enzymes cystathionine γ-synthase (CGS, EC 2.5.1.48) and cystathionine β-lyase (CBL, EC 4.4.1.8) (Figure 1A). These organisms also demonstrate de novo production of cysteine via a sulfur assimilation mechanism in which serine acetyltransferase (SAT, EC 2.3.1.30) produces O-acetylserine (OAS) from acetyl-coenzyme A and l-serine. Next, cysteine synthase (CS, OAS thiolyase, EC 2.5.1.47) catalyzes a β-substitution reaction of OAS with sulfide to produce cysteine. Subsequently, CGS converts cysteine into cystathionine, which is hydrolyzed to homocysteine by CBL (Figure 1A).

Parasitic protozoa are characterized by remarkable differences in the above-described transsulfuration pathways. For example, *Entamoeba histolytica* does not possess genes encoding enzymes of either the reverse or forward transsulfuration pathways [2]. The reverse transsulfuration enzymes CBS and CGL are absent in *Plasmodium falciparum*, *Giardia duodenalis*, *Cryptosporidium parvum*, and *Trichomonas vaginalis* [2,3,4,5]. The de novo pathway has been found in *Entamoeba histolytica* and *Entamoeba dispar* [6]. In *Trypanosoma cruzi*, the enzymes CBS, CGL, and CS have been identified and characterized [7,8], indicating that in this pathogen cysteine might be produced by de novo synthesis processes, as well as by the reverse transsulfuration pathway.

The presence of genes for the enzymes CBS (TGME49_059180), CGL (TGME49_112930), and CS (TGME49_078910) in the genome of the protozoan parasite *Toxoplasma gondii*, the causative agent of toxoplasmosis in humans, raises the possibility that this parasite possesses both the reverse transsulfuration and de novo cysteine biosynthetic routes. However, even if sequence analysis can provide key information on the function of a gene product, further enzymatic and biochemical characterization of the encoded protein is necessary to unequivocally identify its function and properties. Moreover, since the main transsulfuration enzymes belong to the same so-called fold-type I of PLP-dependent enzymes [9], gene function prediction and gene nomenclature assignment based only on phylogenetics and sequence alignment are often complicated. Importantly, recent research exploring the proteome of *T. gondii* has shown that the three enzymes CBS, CGL, and CS are specifically expressed in the oocyst/sporozoite stage, which plays major role in the transmission of the parasite to new hosts and ecosystems [10,11].

The three-dimensional structures of human and yeast CGL have recently been solved using X-ray crystallography [12,13]. CGLs are homotetramers with a subunit molecular mass of approximately 45 kDa and one PLP cofactor per monomer covalently bound through a Schiff base to an active-site lysine. The initial step in CGL reaction mechanism includes transaldimination, in which the α-amino group of the l-cystathionine substrate replaces the ε-amino moiety of the active-site lysine to form the external aldimine. The α-proton is subsequently abstracted, forming a quinonoid intermediate, which is protonated at the C4′ position to produce a ketimine intermediate. This species undergoes β-proton abstraction with subsequent release of l-cysteine product, and removal of the C4′ proton yielding a quinonoid intermediate, which subsequently undergoes electron rearrangement and protonation at the Cγ position to produce the internal aldimine of aminocrotonate [13,14]. Non-enzymatic hydrolysis of the aminocrotonate intermediate produces α-ketobutyrate and ammonia in aqueous solution (Figure 1B).

In addition to the synthesis of l-cysteine from l-cystathionine via α,γ-elimination, human CGL is largely involved in the α,β-elimination of l-cysteine to produce H_2_S, which has emerged as an important gaseous signaling molecule with roles in various diseases [15]. Moreover, in humans, deficiency of CGL causes the autosomal recessive disease cystathioninuria.

Herein, we have cloned, expressed in *E. coli*, and characterized the putative CGL from *T. gondii* (TgCGL) with the aim to expand the very limited knowledge on the reverse transsulfuration pathway in this pathogen. We show that recombinant TgCGL is functional and splits l-cystathionine (l-cth) almost exclusively at the CγS bond, likely implying that cysteine could be generated via the reverse transsulfuration pathway. Moreover, using in silico modelling and in vitro mutational analysis we identify critical residues within the active site of TgCGL that are involved in modulation and control of reaction specificity.

## 2. Results

### 2.1. Properties of Recombinant TgCGL

The putative TgCGL enzyme is encoded by a single gene (TGME49_112930) and displays the highest sequence identity with CGLs from Trypanosomes and Leishmania parasites (~58% identity). However, it showed also ~40% and ~37% sequence identity with the human and *Saccharomyces cerevisiae* counterparts, respectively (Figure S1).

**Figure 2:**
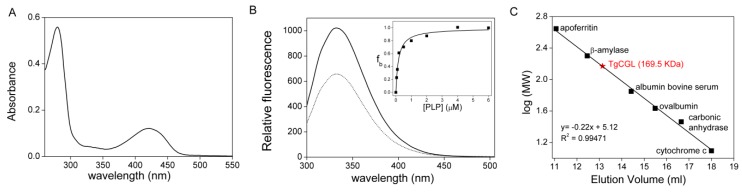
Properties of recombinant TgCGL. (**A**) Absorption spectrum of purified TgCGL recorded in a solution containing 12 µM protein in 50 mM MBP buffer pH 9. (**B**) Fluorescence spectra (excitation was at 280 nm) of holo-TgCGL (dotted line) and apo-TgCGL (solid line) in 50 mM Bis-Tris-propane pH 9, at a concentration of 1 µM. (**Inset**) Representative titration of apo-TgCGL with PLP. The apo-protein was incubated with PLP at different concentrations for 15 min at 25 °C in 50 mM Bis-Tris-propane pH 9 before determination of the fluorescence emission variations at 332 nm upon excitation at 280 nm. The smooth curve shows the best fit to the data with *K*_d_ = 0.17 ± 0.04 µM. (**C**) Analytical gel filtration chromatography of TgCGL. The calibration curve used to estimate the native molecular weight based on the elution position during analytical gel filtration is indicated.

Recombinant native TgCGL exhibited an absorption spectrum with a major peak at 421 nm (Figure 2A). This peak indicates that the predominant tautomer of the internal aldimine is the ketoenamine that PLP forms with Lys230 in the active site (Figure 1B) [16,17].

Determination of enzyme-bound PLP indicated that the TgCGL binds ~1 mol of PLP/mol of monomer, and the dissociation constant (*K*_d_) for the binding of PLP to TgCGL was 0.17 ± 0.04 µM, as measured by titrating apo-TgCGL with increasing amounts of PLP and following the fluorescence signal upon excitation at 280 nm (Figure 2B). Limited trypsin proteolysis experiments on the holo- and apo-forms of TgCGL provided evidence for a protective effect of PLP; in the apo-state TgCGL underwent complete digestion, whereas holo-protein was stable to proteolysis for more than 120 min (Figure S2A,B). Moreover, to investigate if PLP could affect the thermal stability of the enzyme, we determined the melting temperature (*T*_m_) of holo- and apo-TgCGL by differential scanning calorimetry (DSC). The holo-enzyme exhibited higher thermal stability (*T*_m_ of 71 ± 1 °C) (Figure S2C) compared to the apo-protein (*T*_m_ of 55 ± 2 °C) (Figure S2D); therefore, the apo-to-holo transition is associated with a significant increase of the protein thermal stability. Analytical size exclusion chromatography showed that in its native form, TgCGL displayed a molecular mass of ~170 kDa (Figure 2C), indicating that TgCGL is a homotetramer in solution, consistent with a subunit molecular mass of 46 kDa.

### 2.2. Kinetic Properties of TgCGL

**Figure 3:**
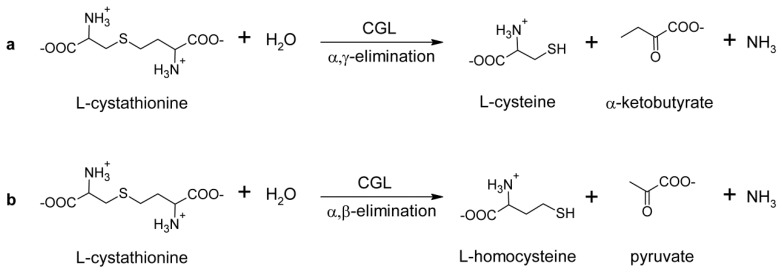
Overview of the α,γ- (**a**) versus α,β-elimination (**b**) reactions of L-cth catalyzed by CGL.

l-cth hydrolytic activity of TgCGL was measured via both the DTNB and LDH assays. Indeed, the DTNB-based assay does not distinguish between the products of l-cth hydrolysis, which are l-homocysteine (l-hcys) and l-cys for the β- and γ-elimination reactions, respectively (Figure 3). Therefore, we also employed a LDH coupled enzyme assay, which possesses a high catalytic efficiency for pyruvate, to specifically follow the α,β-elimination reaction (Figure 3).

To define the appropriate reaction conditions for the enzyme, we firstly determined the effect of pH (in MBP buffer in the pH range 6–10) and temperature (between 20–70 °C) on l-cth hydrolysis of TgCGL. The purified enzyme revealed optimal activity at pH 9 and at 40 °C (Figure S3A,B). Moreover, the enzyme exhibits relatively high thermal stability with a T_50_ value of 64 ± 2 °C (Figure S3C).

Determination of l-cth hydrolysis via DTNB assay at pH 9 resulted in *k*_cat_ of 2.0 ± 0.1 s^−1^ and *K*_m_ of 0.9 ± 0.1 mM (Table 1), whereas negligible activity was detected via LDH-coupled assay (the enzyme specific activity was <0.4% compared to that measured by DTNB assay). Thus, TgCGL was highly specific for α,γ-elimination of l-cth.

**Figure 4:**
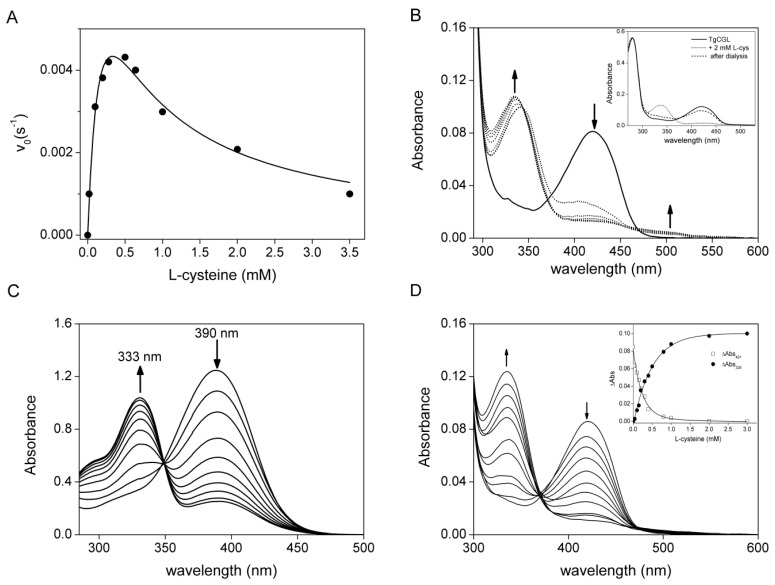
TgCGL and l-cys. (**A**) Representative curve for substrate inhibition of TgCGL by l-cys. The enzyme was incubated with increasing concentrations of l-cys, and pyruvate production was measured by the spectrophotometric assay described in Section 4.2. Changes in initial velocity were plotted as a function of l-cys concentration, and the data were fitted to Equation (1). (**B**) UV-visible absorption spectra of TgCGL upon addition of 2 mM l-cys. Seven spectra are displayed at 0, 5, 10, 20, 40, 50, and 60 min. (**Inset**) UV-visible absorption spectra of TgCGL alone (solid line), upon 1 hr incubation with 2 mM l-cys (dotted line), and following dialysis (dashed line). (**C**) UV-visible absorption spectra of 200 µM PLP with 1 mM l-cys. The spectra were recorded at 5 min intervals for 40 min at room temperature. The reaction mixture contained 50 mM MBP buffer pH 9. (**D**) UV-visible absorption spectra of TgCGL upon addition of increasing l-cys concentrations (0–3 mM). (**Inset**) Absorbance changes at 335 nm (solid circle) and 421 nm (open square) plotted against l-cys concentration. The solid lines represent the fitting to Equation (3).

TgCGL can also utilize djenkolic acid (*k*_cat_ 0.24 ± 0.01 s^−1^ and *K*_m_ 0.51 ± 0.01 mM), and amino-ethyl-l-cysteine, to a lesser extent (*k*_cat_ 0.037 ± 0.001 s^−1^ and *K*_m_ 1.3 ± 0.1 mM), via an α,β-elimination reaction (Table 1). l-cys served as a very poor substrate with a maximum velocity less than 1% of the value of the natural substrate l-cth. Substrate inhibition was also observed (Table 1 and Figure 4A).

### 2.3. Spectral Characterization of the Interaction between TgCGL and l-cys

The availability of large amounts of the recombinant protein allowed us to directly analyze the interaction between TgCGL and l-cys by collecting absorption spectra in order to understand why inhibition occurred at higher concentrations of l-cys (Figure 4A). Addition of 2 mM l-cys to TgCGL resulted in an immediate decrease and broadening of the 421 nm peak with the concomitant appearance of new maxima at 335 and 505 nm. No further changes in this UV-visible spectrum were observed when the sample was incubated for ~1 h (Figure 4B). The apparent spectral changes of TgCGL in the presence of l-cys clearly indicate the interaction of l-cys with enzyme-bond PLP. l-cys can enter into the active site of the enzyme, resulting in the formation of cysteine-PLP external aldimine, which next undergoes intramolecular cyclization to form a thiazolidine ring between the formyl group of PLP and sulfhydryl groups of l-cys. We suggest that the 335 nm peak could represent the formation of a thiazolidine adduct, as already observed for human and yeast CGL [18,19] and for other PLP-dependent enzymes [20]. This finding may offer an explanation for the inhibitory effect of l-cys. Addition of l-cys to free PLP led to progressive 390-nm peak decrease and 335-nm peak increase that resulted in the changes observed for TgGCL in the presence of l-cys, except that the peak for TgCGL was at 421 nm instead of 390 nm (Figure 4C) [20,21,22]. Notably, after dialysis of the enzyme thiazolidine complex, the 335 nm peak decreased and the 421 nm peak was regenerated, indicating that thiazolidine formation is reversible (Figure 4B, inset). Reversibility of thiazolidine formation has been reported previously [18,20,21,23,24,25].

We also collected the spectra of enzyme upon addition of increasing concentrations of l-cys (Figure 4D). The plots of the absorbance changes at 335 and 421 nm against the l-cys concentration (Figure 4D, inset), fitted to Equation (3), yielded equilibrium dissociation constant for TgCGL-l-cys complex formation *(K*_app_) values of 306 ± 41 µM and 273 ± 52 µM, respectively.

### 2.4. Molecular Modelling

We modelled the external aldimine of the wild type enzyme in complex with l-cth, starting from the homology-modelled tetramer structure of TgCGL (residues 35–417) in its internal aldimine state. To this end, we used the crystal structures of methionine γ-lyase from *Citrobacter freundii* (PDB: 5E4Z; % identity ≈ 44.2) and human CGL (PDB: 3ELP; % identity ≈ 41.3) as structural templates.

**Figure 5:**
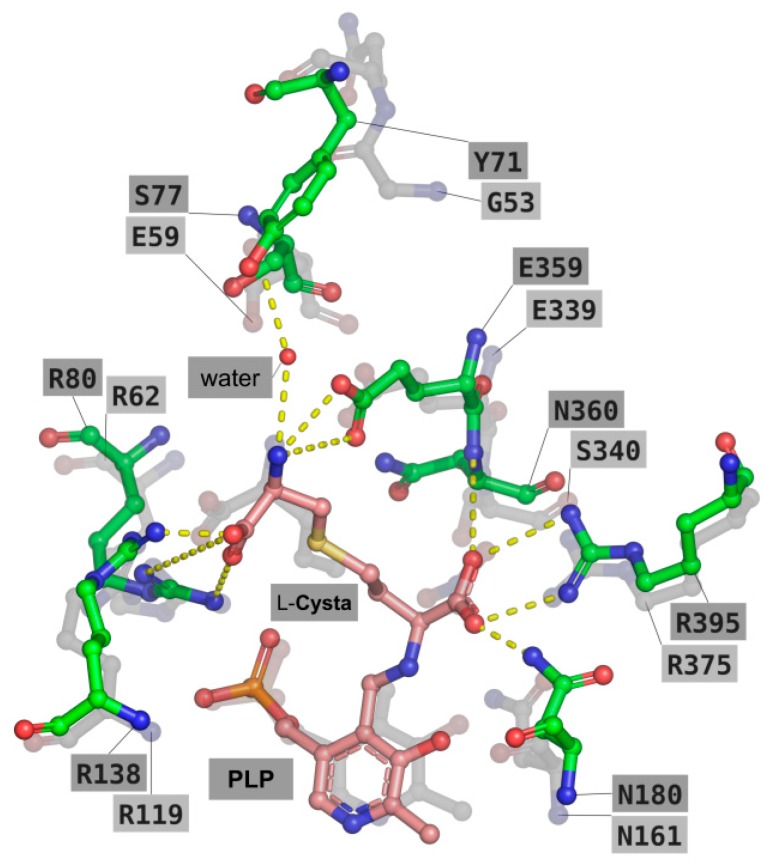
Modelling of the external aldimine of wild type TgCGL in complex with l-cth. TgCGL is shown as green sticks and is superposed to human CGL (PDB: 3COG; transparent gray sticks). The external aldimine is shown as pink sticks. Residues described in text are labeled in single-letter code. Potential favorable interactions are depicted as yellow. Other atoms are colored according to the following scheme: oxygen, red; nitrogen, blue; sulfur, yellow; phosphorus, orange.

The predicted binding mode of l-cth in wild type TgCGL is shown in Figure 5. With the α-amino group of the substrate covalently bound to PLP, the carboxylate group of l-cth is well positioned to form an ion-pair with Arg395 and hydrogen-bonds with Asn180 and the N main-chain atom of Asn360. These interactions are also well conserved in the crystal structure of human CGL (residues Arg375, Asn161, Ser340; PDB: 3COG), in which a nitrate ion occupies approximately the same position of the carboxylate group of l-cth in the predicted docked model. Moreover, and similar to what observed in the crystal structure of human CGL, the ζ-amino and ζ-carboxyl groups of l-cth were predicted to share the same interactions with two arginine residues (i.e., Arg80 and Arg138 of TgCGL and Arg62 and Arg119 in human CGL) and a glutamic acid (i.e., Glu359 of TgCGL and Glu339 in human CGL). Another interaction involving the ζ-amino moiety is represented by a water-bridged hydrogen bond with the hydroxyl group of Tyr71. The latter residue has no homologous counterpart in the human CGL, and its water-bridged hydrogen bond interaction with l-cth is replaced by the carboxyl group of human CGL Glu59.

### 2.5. Deciphering the Importance of Asn360 and Ser77 of TgCGL on Activity and Stability

When comparing the modelled position of PLP-l-cth and its interacting residues in the active site of TgCGL with the corresponding residues in human and yeast CGLs, one striking difference is the replacement of the conserved residues of Ser334/Ser340 and Glu48/Glu59 (numbering refers to the yeast and human CGL, respectively) with Asn360 and Ser77 in TgCGL (Figure 5). Therefore, we decided to replace these two positions in TgCGL with the corresponding residues of human and yeast enzymes to test their effect on enzyme activity.

#### 2.5.1. N360S Variant

**Figure 6:**
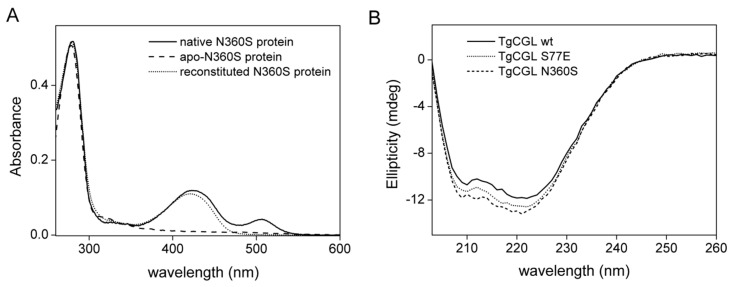
Properties of TgCGL N360S variant. (**A**) UV–visible absorption spectra for native N360S enzyme after purification (solid line), apo-protein (dashed line), and reconstituted holo-enzyme (dotted line). (**B**) Far-UV CD spectra of TgCGL wild type (solid line), S77E (dotted line) and N360S (dashed line) variants.

The absorbance spectrum of N360S showed a major peak at 421 nm, as the wild type protein, and a minor peak at ~500 nm, which is not present in wild type enzyme but previously seen in the human CGL protein preparations [12,14,26]. This 500 nm absorption band disappeared after reconstitution of TgCGL N360S, with PLP following the generation of apo-protein (Figure 6A). It is therefore tentative to speculate that it could be ascribed to a relatively stable quinonoid PLP derivative formed by a bound amino acid that co-purifies with TgCGL. As for the wild type enzyme, the N360S variant binds ~1 mol of PLP/mol of monomer. The mutation did not alter the conformation of the protein respect to the wild type, as evident from far-UV CD spectra (Figure 6B).

Substitution of N360 with Ser led to a ~56-fold decrease in catalytic efficiency for l-cth hydrolysis when analyzed via DTNB assay compared to the wild type enzyme, a change that was influenced by a matched ~10-fold decrease in *k*_cat_ and ~7-fold increase in *K*_m._ Most importantly, α,β-cleavage of l-cth estimated via LDH-based assay was significantly increased (from negligible activity <0.4% of that detected by DTNB assay in the wild type enzyme to ~33% in N360S variant). Moreover, the *K*_m_ for β-elimination of l-cth was reduced by ~6-fold compared to that obtained by DTNB analysis (Table 1), suggesting that the binding of l-cth in a conformation suitable for α,β-elimination is favored.

Qualitative comparison of the reaction products with l-cth, l-hcys, and l-cys standards via thin layer chromatography (TLC) analysis confirmed the production of both l-hcys and l-cys when l-cth was used as the substrate for the N360S enzyme versus the wild type enzyme, for which the primary product is cysteine. This finding confirmed the ability of the N360S variant to catalyze both α,β and α,γ-elimination towards l-cth (Figure S4).

The replacement of Asn360 by Ser had no significant effect on α,β-elimination activity towards l-cys and djenkolic acid, since for these substrates the enzyme variant showed near wild type kinetic parameters (Table 1). l-cys behaves like a poor substrate also of TgCGL N360S, and is characterized by a substrate inhibition profile with an inhibition constant (*K*_i_) of ~1 mM. The *K*_app_ for TgCGL N360S-l-cys complex formation determined from spectral analyses was 335 ± 15 µM at 335 nm and 331 ± 20 µM at 421 nm (Figure S5A).

**Figure 7:**
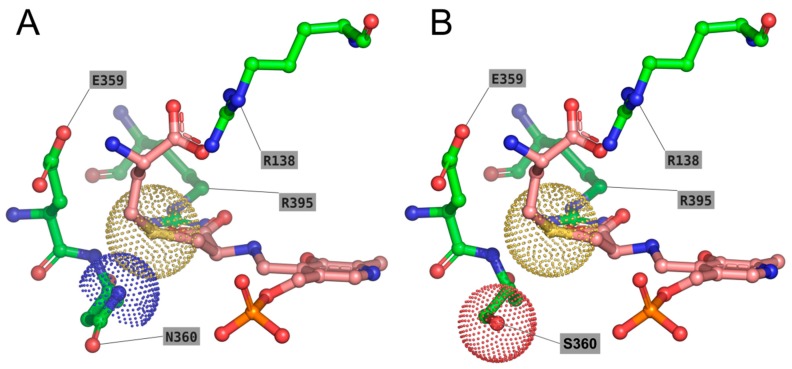
Modelling of the external aldimine of PLP-l-cth in complex with wild type TgCGL (**A**) and N360S mutant TgCGL (**B**). The external aldimine of PLP-l-cth is shown as pink sticks, and the surrounding residues as green sticks. Residues described in text are labeled in single-letter code. The Van der Waals radius of the Sγ atom of l-cth is shown as yellow dots. The Van der Waals radii of Nγ N360 and Oβ S360 are shown as blue and red dots, respectively.

The modelled N360S enzyme in its external aldimine form in complex with the l-cth substrate bound in opposite orientation (in agreement with the distinct α,β versus α,γ-elimination reaction specificity requirements, i.e., S atom in γ or δ position, respectively) allowed us to rationalize the behavior of TgCGL N360S. Notably, in the presence of N360, the binding of l-cth in a conformation suitable for α,β-elimination is partially hindered (see Figure 7A and compare with the orientation of l-cth shown in Figure 5), due to the slight steric clash with the bulky S atom in position γ of l-cth. On the contrary, the N360S mutant is predicted to accommodate well both binding modes of l-cth (Figure 7B).

#### 2.5.2. S77E Variant

We found no difference in the absorption of S77E variant or in the stoichiometry of PLP-bound (~1 mol of PLP per monomer) compared to the wild type enzyme (Figure S6).

Notably, replacement of Ser77 by glutamate completely abolished its activity toward l-cth. We made different attempts to determine enzymatic activity, including long incubation times and increased substrate and enzyme concentrations, but no activity was found with either the DTNB or LDH assay. In addition, the α,β-elimination activity of TgCGL towards djenkolic acid and amino-ethyl-l-cys was heavily compromised resulting in a 213- and 90-fold decrease in catalytic efficiency, respectively, compared to wild type enzyme (Table 1). The analysis of the interaction between TgCGL S77E and l-cys by absorption spectra resulted in a *K*_app_ of 677 ± 180 µM at 335 nm, and 874 ± 87 µM at 421 nm for TgCGL S77E-l-cys complex formation (Figure S5B). Through CD studies, we ascertained that the loss of enzyme activity for the mutant was not due to disruption of overall protein structure (Figure 6B). The potential of the active-site residue S77 to influence l-cth hydrolysis was also explored by characterization of the S77A substitution. However, the near wild type kinetic parameters for S77A variant did not support a direct and key role for this residue in catalysis (Table 1).

**Figure 8:**
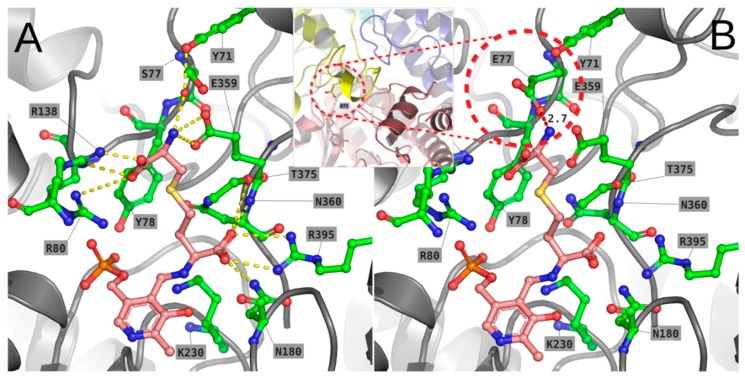
Modelling of the external aldimine of wild type (**A**) and the S77E mutant (**B**) of TgCGL in complex with l-cth. The external aldimines are shown as pink sticks. Residues described in text are labelled in single-letter code. Potential favourable and unfavourable interactions are depicted as yellow (**A**) and red (**B**) dashes, respectively. The position of the loop hosting E77 relatively to the other chains (A = yellow; B = pink; C = cyan; D = blue) of the quaternary structure is shown in the inset of panel (**B**).

Due to the presence of Tyr71, which is missing in human CGL, in the modelled S77E mutant the side-chain of Glu77 is unable to occupy the same position of the homologous Glu59 of human CGL, since this would cause a steric clash with Tyr71 (Figure 8). Instead, the side-chain of Glu77 is forced to occupy the same cleft of l-cth, possibly interfering with its binding. The overall effect of the S77E mutation could also affect the stability of the enzyme, since this residue is placed on a loop (residues 70–78), making close contacts with two other monomers of TgCGL (i.e., residues 354–360 of the chain forming the dimeric unit, and residues 46–56 of the loop coming from the other dimer). In particular, it is expected that a local perturbation of such contacts, due to the S77E mutation, could weaken the interaction between the two dimers composing the tetrameric structure (Figure 8, inset window).

**Figure 9:**
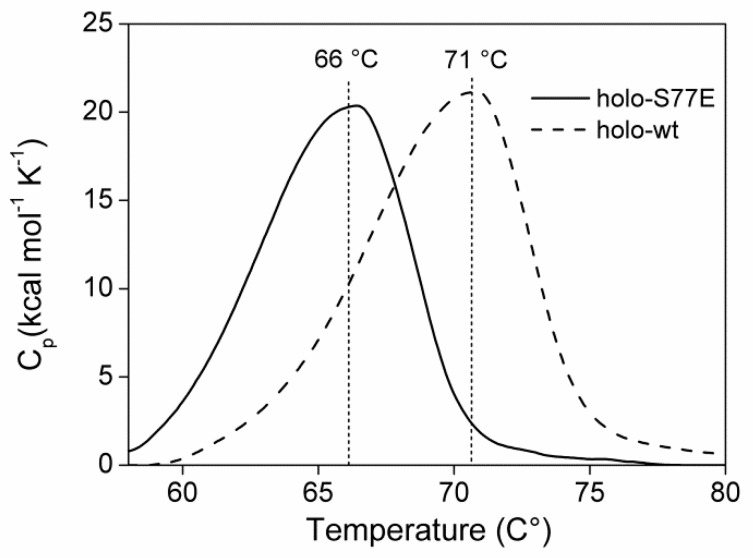
Effect of S77E mutation on thermal stability of TgCGL. Representative DSC thermograms of native S77E (solid line) and wild type (dashed line) protein. DSC traces are baseline-corrected.

The impact of the mutation on structure stability of TgCGL was probed by analyzing the thermal stability of the protein by DSC. Comparative analyses between wild type and variant proteins were performed at equal scan rates on samples containing identical protein concentrations. To avoid the assumptions or simplifications inherent in model-based analyses, we limited our investigation to a phenomenological analysis of the experimental data. Indeed, the midpoint of thermal unfolding curves provides an adequate indication of the relative thermal stability of related proteins [27]. DSC data showed a decreased apparent thermal transition midpoint of S77E variant (*T*_m_ = 66 ± 1 °C) compared to wild type protein (*T*_m_ = 71 ± 1 °C), therefore indicating that the single S77E amino acid substitution influences protein thermal stability (Figure 9).

## 3. Discussion

The characterization of the enzymes involved in essential metabolic pathways is fundamental for the understanding of parasite biology, and may also provide crucial information for the rational design of novel therapeutics.

The recent finding of a putative oocyst/sporozoite-specific subset of proteins (POSPs) in *T. gondii*, the causative agent of toxoplasmosis, [10] opens new possibilities for the identification of novel candidates for drug development against the parasite. In particular, three POSP enzymes involved in cysteine metabolism (CBS, CGL, and CS) could represent interesting drug targets due to their central metabolic roles. Indeed, l-cys is indispensable for the survival of all living organisms and plays a significant role in maintaining the redox balance of thiol compounds. Therefore, there is the need to expand the knowledge about the functional and structural properties of *T. gondii* enzymes involved in the cysteine biosynthetic pathway.

Herein, we demonstrate that *T. gondii* possesses a functional CGL. The primary reaction catalyzed by TgCGL has been shown to be α,γ-hydrolysis of l-cth to form l-cys, α-ketobutyrate and ammonia. The steady state kinetic parameters of TgCGL for l-cth α,γ-elimination were shown to be very similar to those of *T. cruzi*, another protozoan parasite [8], and *S.* *cerevisiae* [28], with low catalytic efficiency (*k*_cat_*/K*_m_ ~ 2 mM^−1^·s^−1^), whereas the human enzyme possesses higher catalytic efficiency (*k*_cat_*/K*_m_ ~ 15 mM^−1^·s^−1^) [12]. Importantly, CGL from *T. gondii* splits the l-cth substrate almost exclusively at the CγS bond, similar to CGL from *T. cruzi* [8] and human CGL [12,14], while CGL from yeast [28] shows a pronounced CBL-like activity, i.e., it is able to cleave both the CγS and CβS bonds of l-cth. Interestingly, TgCGL exhibits high temperature optimum and high thermal stability for l-cth hydrolysis. This could be attributed to the parasite’s adaptation to a broad range of temperatures during its life cycle. Indeed the parasite, and in particular in the oocyst stage, exists in diverse hosts such as human (36.5–37.5 °C), cattle (36.7–39.1 °C), cats (38.6–40.1 °C), dogs (37.9–39.9 °C), chickens (39.6–43.6 °C), and geese (40.0–44.0 °C). Moreover, parasite infection can cause an inflammatory response accompanied by fever.

In addition to its role in the conversion of l-cth into l-cys, studies on human and yeast CGL have also shown that the enzyme can catalyze the α,β-elimination of l-cys to produce H_2_S [12,14,18,28]. The yeast enzyme attacks the CβS bond of l-cys with a catalytic efficiency very similar to that for the γ-elimination of the natural substrate l-cth [28]. On the contrary, in human CGL and in TgCGL l-cys is converted orders of magnitudes more slowly than l-cth. However, TgCGL displayed only marginal reactivity toward l-cys in comparison to the human enzyme (*k*_cat_*/K*_m_ ~ 0.15 mM^−1^·s^−1^ in human vs. ~0.043 mM^−1^·s^−1^ in toxoplasma), suggesting that TgCGL does not have a significant role in H_2_S production. Along with this, CGL from *T. cruzi* is unable to produce H_2_S [8], whereas human CGL is believed to be chiefly responsible for biogenesis of H_2_S, which is considered an important gaseous signaling molecule with roles in various diseases.

Interestingly, we found that l-cys is characterized by a substrate inhibition profile. It has been reported that, in addition to the general ability to work as a substrate for most CGL enzymes, l-cys may inhibit the enzyme activity by partially removing PLP from the holoenzyme when the concentration of l-cys is higher than a threshold level [12,18,19,23,29]. However, the mechanistic origin for the release of the PLP cofactor is still unknown. Herein, we found that l-cys clearly interacts with TgCGL-bond PLP, likely forming a thiazolidine complex, therefore offering an explanation for its inhibitory effect. However, the absorbance spectrum of the enzyme incubated with l-cys, following dialysis against buffer without PLP, was restored to a near native spectrum of recombinant native TgCGL, suggesting that the holoenzyme can be regenerated and the formation of the thiazolidine is reversible to a large extent. Notably, the absorption spectrum of the yeast CGL upon addition of l-cys showed the immediate formation of a l-cys-derived PLP-thiazolidine adduct by the decrease of the 420 nm absorbing species (internal aldimine) and the concomitant increase of the typical 333 nm peak. However, after only 1 min of incubation, the 333 nm peak (thiazolidine) decreased, and the 420 nm peak was regenerated [18]. Thus, the exact structural and chemical basis for CGL inactivation via thiazolidine formation at high l-cys concentration remains to be clarified. Differences in the cysteine-dependent inactivation could be dependent on the relative position of l-cys and bound PLP at the active site, being likely affected by the nature of key active site residues that may restrict the flexibility of the sulfhydryl group of cysteine from undergoing nucleophilic addition to form a complex with PLP. Thus, the relation between l-cysteine and PLP is very complex and, unfortunately, there have been few detailed investigations of the enzyme with respect to the elimination of cysteine. Moreover, no information is available on the free intracellular levels of cysteine in toxoplasma for comparison with our data. In fact, despite the major human health problems caused by *T. gondii*, many aspects of its biology are still unknown.

Our studies suggest that CGL is an important functional enzyme in *T. gondii*, likely implying that cysteine could be generated via a reverse transsulfuration route. On the other hand, due to the absence of CGS and CBL enzymes, *T. gondii* seems to lack the forward transsulfuration pathway, being unable to accomplish transsulfuration reactions in the direction from cysteine to methionine. Further work is needed to demonstrate the specific role of the other components involved in l-cys biosynthetic pathway, i.e., putative CBS and CS, in order to corroborate the metabolic and physiological importance of the sulfur metabolism.

Due to the involvement of TgCGL in a potential operative reverse transsulfuration pathway, the enzyme could represent a strong candidate for rational inhibitor design. However, the degree of similarity between TgCGL and human CGL (e.g., 40% sequence identity) makes the design of inhibitors that can distinguish between the active sites of the two CGLs a difficult task. Inspection of superposed human and parasite CGL active sites allowed us to identify two prominent differences at position 340 and 59 (human CGL numbering). Sequence analysis indicated that residues such as Ser340 and Glu59 are conserved in CGLs of all organisms except protozoa, e.g., trypanosomes in general, *Lehismania major*, and *Toxoplasma*, in which an Asn and a Ser residue, respectively, are present*.*

Mutation of N360 to the corresponding residue in human (Ser) was found to influence the reaction preference of the parasitic enzyme, such that the N360S mutant harbors l-cth β-lyase activity. Determining the reaction specificity for catalysis of α,β- versus α,γ-elimination of the same substrate l-cth represents an intriguing and compelling issue. The distinct β- versus γ-elimination reaction specificity requires that the pseudo-symmetric l-cth substrate must react with the PLP cofactor in two opposite orientations, with S in the γ or in δ position, respectively [13]. Our findings clearly imply that in TgCGL the Asn residue at position 360 plays an important role in enforcing the appropriate binding conformation of l-cth within the active site, such that S is preferentially placed in the δ-position. Moreover, replacement of TgCGL-N360 with Ser results in a ~6-fold increase in *K*_m_ for hydrolysis of l-cth when assessed via DTNB, confirming the role of the residue as a determinant of reaction specificity via positioning of the l-cth substrate in the orientation required for γ-elimination. The investigation of the active site of TgCGL and the accommodation of l-cth in a conformation suitable for α,β-elimination suggest that the latter is hindered, in part, due to steric clash with the bulky S atom in position γ of l-cth. Replacing Asn360 with Ser, in turn, permits both binding modes of l-cth, with S in γ-position or in δ-position. Accordingly, the S atom of the l-cth substrate was considered as an important factor in orienting substrate binding and, therefore, specificity of the reaction, in yeast CGL [13]. Moreover, the fact that N360S substitution does not affect activity towards β-elimination-specific substrates supports a role of N360 in influencing reaction specificity in the context of TgCGL.

Reaction specificity for the catalysis of α,γ- versus α,β-elimination has been proposed to depend on a pair of highly conserved glutamate residues (E59-E339 in human CGL and E48-E333 in yeast CGL), which are both located at the entrance of the active site and function to coordinate the cysteinyl moiety of l-cth, forming an area with a negatively-charged surface [13,30]. In particular, mutational analysis of E333 revealed a role for this residue in defining the orientation of substrate binding in yeast CGL via electrostatic repulsion with the S atom of l-cth in γ-position, as required for β-elimination, rather than the δ-position, as needed for γ-elimination [28]. Concomitant substitution of both E48 and E333 in yeast CGL selectively impairs l-cth hydrolysis with respect to substrates specific for β-elimination reaction [28]. In human CGL, substitution of the corresponding E339 with a hydrophobic residue increased H_2_S production from l-cys, a substrate that allows only α,β-elimination/replacement [19]. On the other hand, single amino acid substitutions of E48 in yeast CGL do not provide support for a frank role of this amino acid in either reaction specificity or binding of substrate, as they all possess near-wild type kinetic parameters [28]. Replacement of the corresponding D45 of CGS residues from *E. coli* leads to subtle differences in kinetics, while allowing for minor transamination activity. This thus suggests that it has an indirect role in active site geometry or positioning of substrate. Substitution of S77 of TgCGL to Glu, mimicking human and yeast CGL, resulted in a complete loss of enzyme activity toward l-cth. A possible structural explanation of this behavior comes from the observation of the modelled mutant in the active site of TgCGL: the presence of Tyr71, which is missing in human CGL, forces Glu77 to occupy the same cleft of l-cth, possibly interfering with its binding. Every attempt to model Glu77 in a different rotameric position (e.g., replacing Glu359 in its interaction with the substrate’s amino group) resulted in severe clashes of the two glutamate residues with other residues of the active site. We also evaluated the possibility that Tyr71 could adopt a different rotamer to adapt to the presence of Glu77, but the observation of the obtained model strongly suggests that the aromatic ring of Tyr71 is trapped in a hydrophobic cleft formed by the surrounding residues of the helix-loop 68–76. Replacement of Ser77 by Glu also decreases the overall stability of the enzyme, probably perturbing the interaction between the two dimers composing the tetrameric structure, since this residue is placed on a loop, making close contacts with two other monomers of TgCGL.

Altogether, our results reveal the essential role of the specific structural context of TgCGL in defining the properties of the enzyme and provide support for the theory, suggested by Clausen [31], that orientation of substrate in the active site is a crucial determinant of reaction specificity in PLP-dependent enzymes of the γ-subfamily, in addition to freedom of rotation of the Cα–Cβ bond of the substrate during covalent binding to cofactor. Moreover, our findings strengthen the idea that transsulfuration enzymes provide an ideal model system to study the mechanisms whereby enzymes control reaction specificity. Exploration of the underlying factors that enable enzymes to control substrate and reaction specificity will enable engineering of these properties and might have far-reaching implications for the use of transsulfuration enzymes as anti-toxoplasmosis drug targets.

## 4. Materials and Methods

### 4.1. Protein Production

The complete cDNA of TgCGL (accession number: XM_002364464) in pMA-T vector was obtained from Invitrogen Corporation with a tag of six His at the N-terminal. The gene was cloned into the pET21a vector, and various *E. coli* strains and growing temperatures were screened for optimum soluble protein production. The best expression conditions were *E. coli* Rosetta (DE3) cells and overnight induction with 0.5 mM isopropyl-β-d-1-thiogalactopyranoside (IPTG) at 24 °C.

Cells were isolated by centrifugation (5000 *g* for 15 min), resuspended in 20 mM sodium phosphate pH 8, 150 mM NaCl, and 0.1 mM DTT buffer containing 1× protease inhibitor EDTA free (Sigma-Aldrich, S8830, Milano, Italy), and lysed by sonication (12 cycles of 10 s ON/20 s OFF on ice water). After centrifugation at 30,000 × *g* for 30 min at 4 °C, the supernatant was loaded onto an Ni-affinity column previously equilibrated with 20 mM sodium phosphate at pH 8, 150 mM NaCl, 0.1 mM DTT, and 10 mM imidazole. The column was washed with the equilibration buffer until the absorbance at 280 nm reached zero. TgCGL was eluted from the column by applying a gradient of imidazole from 10 to 500 mM. After addition of 100 μM PLP, the fractions containing TgCGL were concentrated and washed with 20 mM sodium phosphate, 0.1 mM DTT buffer, and pH 8, using Vivaspin concentrators (Sartorius, Göttingen, Germany) to remove imidazole and unbound PLP.

The extinction coefficient was used to calculate the monomer concentration of the purified protein (ε_280nm_ = 30745 M^−1^·cm^−1^; http://web.expasy.org/protparam/). The PLP content of the holo-enzyme was determined by addition of 0.1 M NaOH and using ε_388nm_ = 6600 M^−1^·cm^−1^ as described [32,33].

The S77E, S77A, and N360S TgCGL variants were produced by site-specific mutagenesis on the pET21a-TgCGL construct using the QuikChange^®^ site-directed mutagenesis kit (Agilent Technologies, Santa Clara, CA, USA). The primer sequences used are listed in Table S1. The mutated sequence was confirmed by DNA sequence analysis. Expression and purification of the three variants were performed as described for wild type TgCGL. The yield was approximately 20 mg of protein/L of cell culture for all enzyme variants. The homogeneity and purity of enzyme (>95%) was confirmed by SDS/PAGE, and the band size of the recombinant protein, calculated with a molecular size marker, was ~46 kDa, which corresponds well to the predicted molecular mass of the protein.

### 4.2. Enzyme Activity Assays

Enzyme activity was determined using a Jasco-V560 UV-Vis spectrophotometer (Jasco Europe, Cremella (LC), Italy) via two spectrophotometric assays: the reaction of 5,5′-dithiobis-(2-nitrobenzoic acid) (DTNB) with the free thiol of the product (*ε*_412_ = 13600 M^−1^·cm^−1^) and by monitoring the pyruvate formation with the coupling enzyme NADH-dependent lactate dehydrogenase (LDH) (*ε*_340_ = 6200 M^−1^·cm^−1^), as described [34,35]. All enzymatic assays were carried out at 37 °C in 50 mM MOPS, bicine, proline (MBP) buffer pH 9 in the presence of 20 μM PLP. Reactions were initiated by the addition of the enzyme at a final concentration of 1, 5, or 10 μM, depending on the activity of the enzyme. A background reading was obtained prior to initiating the reaction by addition of TgCGL.

Data were fitted to the Michaelis–Menten equation to obtain values of *k*_cat_ and *K*_m_. For site-directed variants for which saturation was not observed, *k*_cat_/*K*_m_ values were obtained by linear regression, based on the assumption that *K*_m_ >> [substrate]. The l-cys hydrolysis data were fitted to Equation (1), which modifies the Michaelis-Menten equation taking into account the *K*_i_ term for substrate (S) inhibition by l-cys [36]:

(1) $$\frac{v}{E}=\frac{k_{\mathrm{cat}}}{1+\frac{K_{m\;}}{S}+\;\frac{S}{K_{i}}}$$

in which *E* is the total enzyme concentration.

To assess the stability of the enzyme at different temperatures, the enzyme was incubated for 10 min at temperatures between 20 and 90 °C and cooled on ice for 5 min, and residual enzymatic activity was determined as described above.

### 4.3. Apo-Protein Preparation

Apo-proteins were obtained following the protocol in [35,37]. The apo-proteins showed no absorption peaks in the region 300–500 nm and no residual activity. The equilibrium dissociation constant for PLP (*K*_d_ was obtained by measuring the fluorescence emission at 332 nm upon excitation at 280 nm in the presence of increasing concentrations of PLP (0.05–10 μM) at 25 °C in 50 mM Bis-Tris propane pH 9 at apo-protein concentration of 1 μM. Fluorescence measurements were carried out on a Jasco FP8200 spectrofluorometer using 5 nm bandwidths for both excitation and emission. The values of fluorescence intensity at 332 nm were plotted as a function of PLP concentration and fit to a hyperbolic binding equation to obtain the dissociation constant *K*_d_. The fraction (*f*_b_) of bound TgCGL is determined from the fluorescence enhancement:

(2) $$f_{b}=\frac{\frac{F}{F_{0}}-1}{\frac{F_{\infty }}{F_{0}}-1}$$

in which *F*_0_ is the intensity of TgCGL measured in the absence of PLP. The value of *F*_∞_, the fluorescence of completely bound TgCGL, is determined through extrapolation of the changes in fluorescence to infinite PLP concentration. At least three independent determinations were carried out.

### 4.4. Size Exclusion Chromatography

Gel filtration chromatography (Superdex 200 HR 10⁄300 GL, GE Healthcare Europe GmbH, Milano, Italy) was used to analyze the oligomeric state of TgCGL. Chromatography was performed using 50 mM sodium phosphate buffer pH 8.5, 150 mM NaCl, 0.1 mM DTT. The calibration curve was obtained as described [38,39,40].

### 4.5. Spectroscopic Measurements

Absorption measurements were performed on a Jasco-V560 UV-Vis spectrophotometer in MBP buffer pH 9, at protein concentration of 12 μM.

For analysis of TgCGL-l-cys interaction, the plots of the absorbance changes at 335 and 421 nm against l-cys concentration were fitted to the hyperbolic Equation (3):

(3) $$\Delta A=\frac{\Delta A_{\max}*\left[cysteine\right]}{K_{\mathrm{app}}+\left[cysteine\right]}$$

in which Δ*A* and Δ*A*_max_ are the absorption changes at given and infinite l-cys concentrations, respectively, and *K*_app_ the equilibrium dissociation constant for TgCGL-l-cys complex formation.

Far-UV (200–260 nm) CD measurements were carried out on a Jasco J-1500 spectropolarimeter using 0.2 mg/mL enzyme in 20 mM sodium phosphate pH 8. Five spectra, averaged automatically, were recorded in a cuvette with a 1 mm path length at a scan speed of 50 nm/min.

### 4.6. Limited Proteolysis

Holo- and apo-TgCGL were treated with trypsin at a 1:200 protease-to-protein ratio (by weight) in 50 mM Tris-HCl pH 7.5 at 25 °C. At various time intervals (0, 1, 5, 10, 20, 40, 60, and 120 min), 8 μL aliquots were taken for electrophoretic analysis. The digestion reaction was ended by boiling the sample for 5 min and adding reducing Laemmli buffer. After staining the gel with Coomassie blue, band intensity analysis was performed as described [41].

### 4.7. Differential Scanning Calorimetry

DSC experiments were carried out with a nano-DSC calorimeter from TA Instruments (New Castle, DE, USA). DSC scans were recorded from 10 to 120 °C at 1 °C min^−1^. Selected experiments were also performed at 0.5 °C min^−1^ to evaluate the scan rate dependence of the thermograms. Samples contained 50–100 μM protein dissolved in 20 mM sodium phosphate pH 8. Corresponding buffer scans were also recorded. All samples were degassed prior to measurements. Analysis was performed using NanoAnalyze (TA Instruments Inc.). The thermal transition midpoint was determined as the temperature corresponding to the peak top.

### 4.8. Thin Layer Chromatography

Amino acid standards (1 mg/mL) were prepared in 20 mM sodium phosphate buffer, pH 8. These solutions were spotted (1 μL) on chromatographic plates comprised of a 0.1-mm thick layer of silica gel on an aluminum support (Merck millipore, TLC Silica gel 60 F254, Darmstadt, Germany) and developed with a mobile phase of n-propanol/water (70:30, *v*/*v*), for a distance of 13 cm. TLC plates were subsequently dried, sprayed with a solution of 2 mg/mL ninhydrin in ethanol, and dried prior to heating for 5 min at 100 °C.

### 4.9. Molecular Modelling Studies

The crystal structure of methionine γ-lyase from *Citrobacter Freundii* in its internal aldimine form (PDB: 5E4Z) and human cystathionine γ-lyase (PDB: 3ELP) were used as a starting point to generate the model of the wild type, N360S and S77E TgCGL enzyme, using the “homology modeling” tool of PyMod 2.0, followed by energy minimization with the BIOPOLYMER package from InsightII (V.2000, MSI, Los Angeles, CA, USA), as already described [12,42,43].

The Dundee PRODRG2 Server [44] was used to build the energy-minimized three-dimensional structure of the PLP-l-cth external aldimine complex, which was then docked into the active site of wild type form of TgCGL using the template-based molecular docking approach of Molegro Virtual Docker (MVD, version 6.0) software (CLCbio^®^). Flexible torsions of the external aldimines were automatically detected by MVD and manually checked for consistency. A search space of 15 Å radius, centered on the active site cavity, was used for docking. The PLP in its internal aldimine form, as found in 3COG, was taken as pharmacophoric group for template-based docking. In the latter, if an atom of the ligand matches a group definition, it is rewarded by using a weighted score that depends on its distance to the group centers. The grid-based MolDock score with a grid resolution of 0.30 Å was used as scoring function, and MolDock SE was used as docking algorithm [45]. For each ligand, ten runs were defined. Six similar poses with RMSD ≤1.0 Å were clustered, and the best scoring one was taken as representative. Other docking parameters were fixed at their default values. After docking, energy optimization of hydrogen bonds was performed.

### 4.10. Statistical Analysis

Each experiment was performed at least in triplicate, and reported values are representative of two or more independent determinations using different batches of protein that were purified separately. Data were analyzed using Origin 8.0 (OriginLab Corporation, Northampton, MA, USA) and expressed as mean ± S.E.M.

## Figures and Tables

**Table 1:** Steady-state kinetic parameters of TgCGL variants ^a^ at pH 9 and 37 °C.

	Assay	*k*_cat_ (s^−1^)	*K*_m_ (mM)	*K*_i_^l-cys^ (mM)	*k*_cat_/*K*_m_ (M^−1^·s^−1^)
**Hydrolysis of l-cystathionine**					
**wild type**	DTNB	2.0 ± 0.1	0.9 ± 0.1	-	(2.2 ± 0.4) × 10^3^
**N360S**	DTNB	0.24 ± 0.02	6.1 ± 1.2	-	(3.9 ± 1.1) × 10
	LDH	0.08 ± 0.01	1.1 ± 0.1	-	(7.3 ± 1.6) × 10
**S77E**	LDH/DTNB	n.d.^b^	n.d. ^b^	-	-
**S77A**	DTNB	0.7 ± 0.1	2.4 ± 0.3	-	(2.9 ± 0.8) × 10^2^
**Hydrolysis of djenkolic acid**					
**wild type**	LDH	0.24 ± 0.01	0.51 ± 0.01	-	(4.7 ± 0.3) × 10^2^
**N360S**	LDH	0.19 ± 0.01	0.86 ± 0.03	-	(2.2 ± 0.2) × 10^2^
**S77E**	LDH	n.s. ^c^	n.s. ^c^	-	2.2 ± 0.6
**S77A**	LDH	0.22 ± 0.02	1.5 ± 0.2	-	(1.5 ± 0.3) × 10^2^
**Hydrolysis of l-cysteine**					
**wild type**	LDH	0.009 ± 0.001	0.21 ± 0.1	0.53 ± 0.02	(4.3 ± 2.1) × 10
**N360S**	LDH	0.007 ± 0.001	0.4 ± 0.1	1.11 ± 0.04	(1.8 ± 0.7) ×10
**S77E**	LDH	n.d. ^b^	n.d. ^b^	-	-

^a^ TgCGL at 1, 5, or 10 μM was used, depending on the activity of the specific enzyme variant. ^b^ Not detected. ^c^ The notation n.s. indicates that S77E does not display saturation kinetics within the solubility limit of the djenkolic acid substrate, such that *k*_cat_/*K*_m_ was determined via linear regression with the assumption that *K*_m_ >> [djenkolic acid].

## Supplementary Materials

Supplementary materials can be found at http://www.mdpi.com/1422-0067/19/7/2111/s1.

## Abbreviations

CGL	Cystathionine γ-lyase
TgCGL	Cystathionine γ-lyase from *Toxoplasma gondii*
CBS	Cystathionine β-synthase
CS	Cysteine synthase
l-cth	l-Cystathionine
l-cys	l-Cysteine
l-hcys	l-Homocsyteine
DTNB	5,5′-Dithiobis-2-nitrobenzoic acid
LDH	NADH-dependent lactate dehydrogenase
TLC	Thin layer chromatography
DSC	Differential Scanning Calorimetry
CD	Circular Dichroism
PLP	Pyridoxal-5′-phosphate
PDB	Protein Data Bank
*T*_m_	Melting Temperature
*K*_m_	Michaelis-Menten constant
*k*_cat_	Turnover rate
*K*_i_	Inhibition Constant
*K*_d_	Dissociation Constant
