# Supplementary material for: Functional Characterization and Structure-Guided Mutational Analysis of the Transsulfuration Enzyme Cystathionine γ-Lyase from Toxoplasma gondii
Source: Int J Mol Sci. 2018 Jul 20;19(7):2111. doi: 10.3390/ijms19072111 (PMC6073527; doi:10.3390/ijms19072111)
Supplement: Supplementary file 1 [file ijms-19-02111-s001.pdf]

International Journal of
Molecular Sciences

Functional Characterization and Structure-Guided Mutational Analysis of The Transsulfuration Enzyme Cystathionine γ-Lyase from *Toxoplasma gondii*

Supplemental Figures

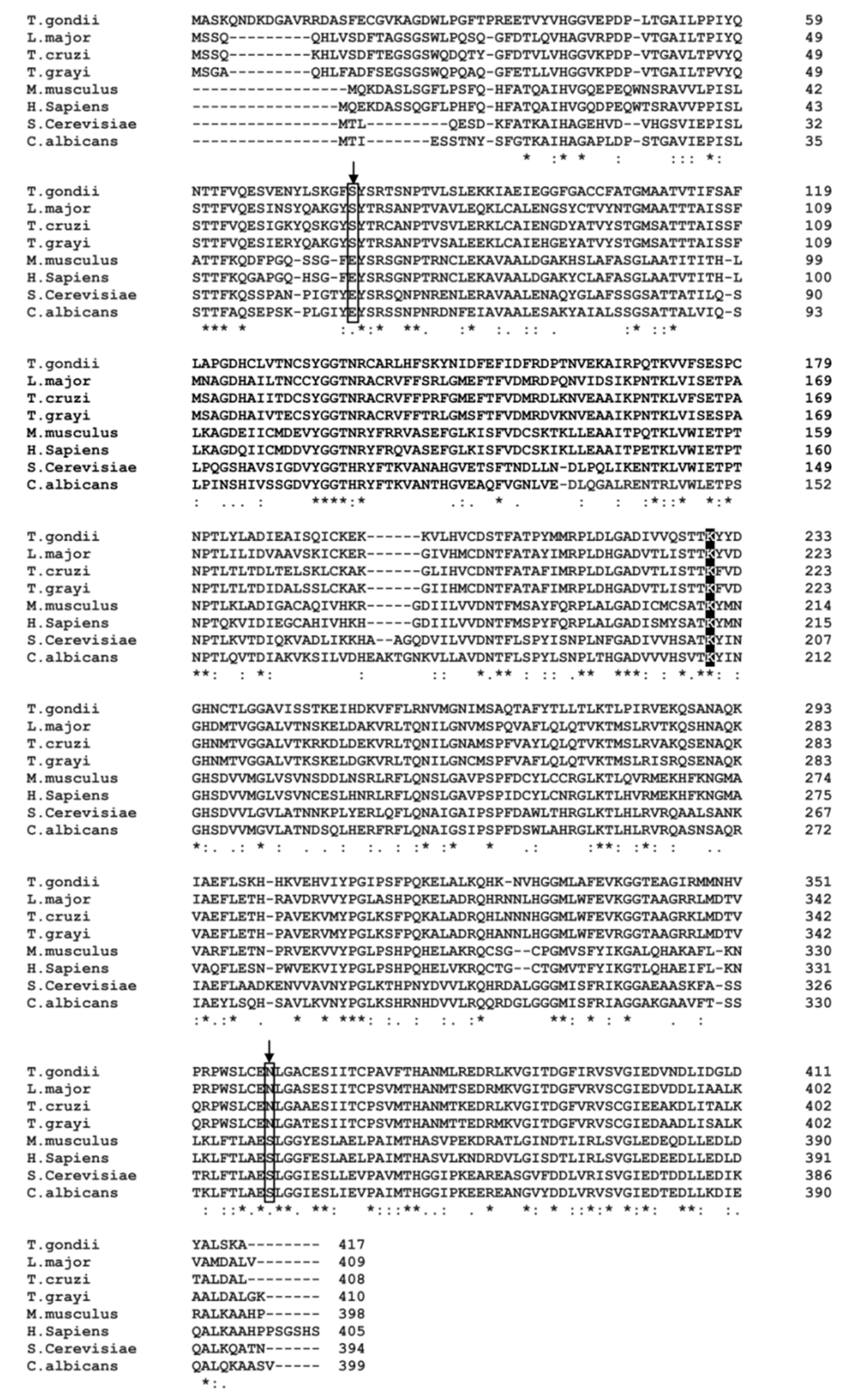

Figure S1. Sequence alignment of CGL from different organisms. Black shading indicates the PLP-binding lysine in the active site. The target residues for mutational analysis are indicated by arrows. The CGLs used in this alignment (NCBI accession number) are XP_002364505.1, *Toxoplasma gondii* ME49; XP_003722717.1, *Leishmania major*; EKG03141.1, *Trypanosoma cruzi*; XP_009313447.1, T*rypanosoma grayi*; NP_666065.1, *Mus musculus*; NP_001893.2, *Homo sapiens*; NP_009390.1, *Saccharomyces cerevisiae*; XP_716241.1, *Candida albicans*. All sequence alignments were carried out using the Clustal OMEGA program.

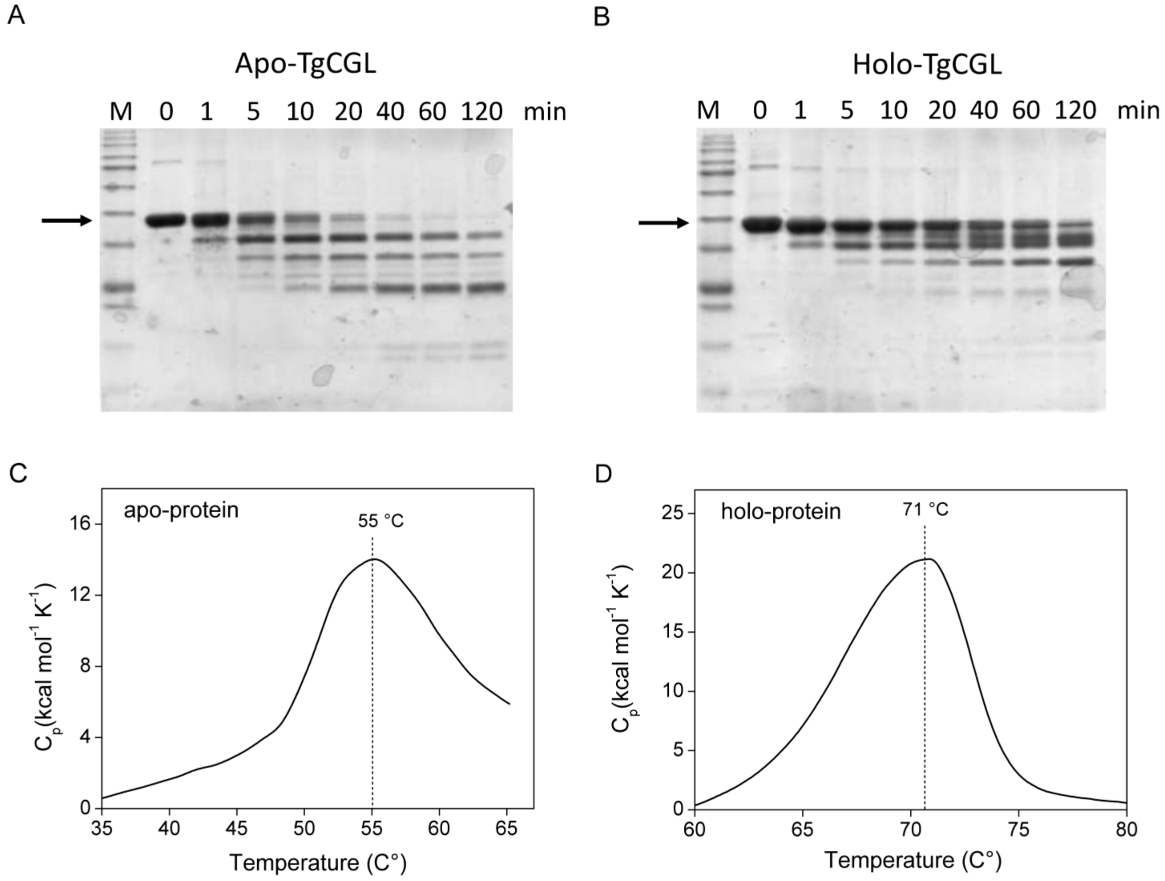

Figure S2. Properties of apo-TgCGL. (**A**) and (**B**). Trypsin digestion profile of apo- (A) and holo-TgCGL (B) after incubation of TgCGL with trypsin 1:200 (*w/w*) for 0, 1, 5, 10, 20, 40, 60 and 120 min, respectively. The intensity of the untreated with trypsin TgCGL band (lane 0 min) was assumed as 100%. The arrow indicates the untreated 46 kDa band. Lane M represents a molecular mass marker. **(C)** and (**D).** Representative DSC thermograms of apo- (C) and holo-TgCGL (D), respectively, after baseline-correction.

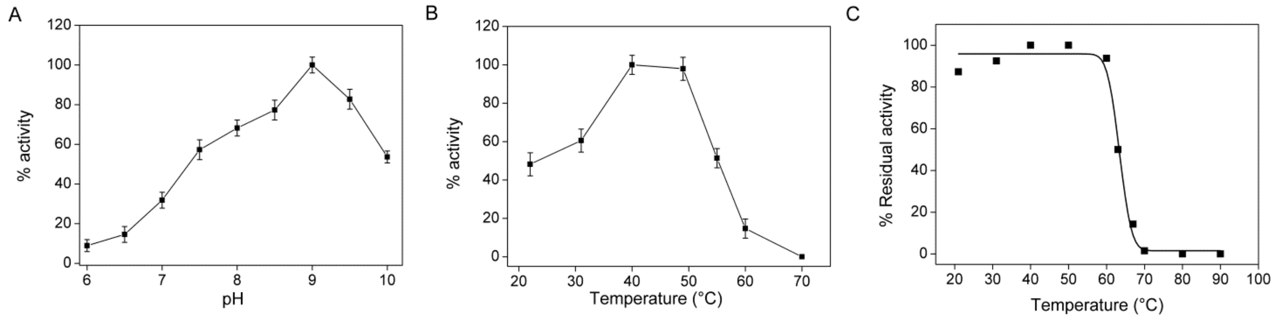

Figure S3. pH and temperature optima for L-cystathionine hydrolysis of TgCGL. (**A**) Effect of pH on purified TgCGL in MBP buffer in the pH range of 6–10 at 37 °C. (**B**) Effect of temperature on the activity of purified TgCGL at pH 9. (**C**) Representative thermal stability curve for purified TgCGL,

held at temperature range from 20 to 90 °C for 15 min. L-cystathionine hydrolysis was estimated using the DTNB assay.

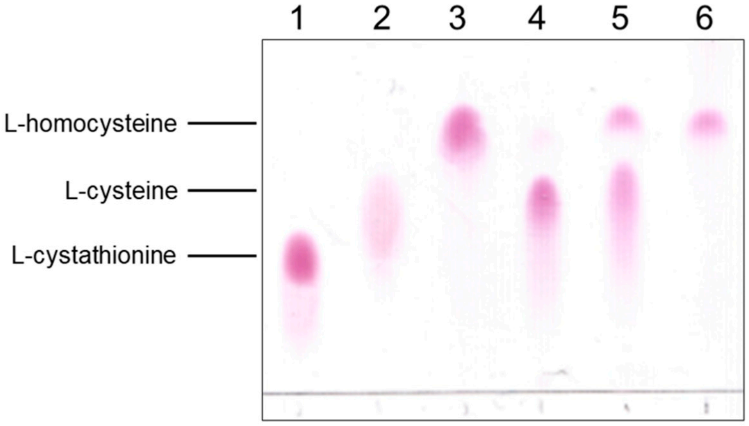

Figure S4. TLC analysis of the amino acid products of L-cystathionine hydrolysis by wt and N360S TgCGL variants. Reaction products and amino acid standards were separated by TLC and derivatized with ninhydrin. Spotting volume is 1 μL. Lane 1–3, amino acids standards. Lane 1: 1 mg/mL L-cystathionine; lane 2: 1 mg/mL L-cysteine; lane 3: 1 mg/mL L-homocysteine; lane 4: 5 mM L-cystathionine + 100 μM wt TgCGL; lane 5: 5 mM L-cystathionine + 100 μM N360S TgCGL; lane 6: 5 mM L-cystathionine + 10 μM cystathionine β-lyase from *Corynebacterium diphtheriae* which catalyzes the β-elimination of L-cystathionine to generate ammonia, pyruvate, and homocysteine [34].

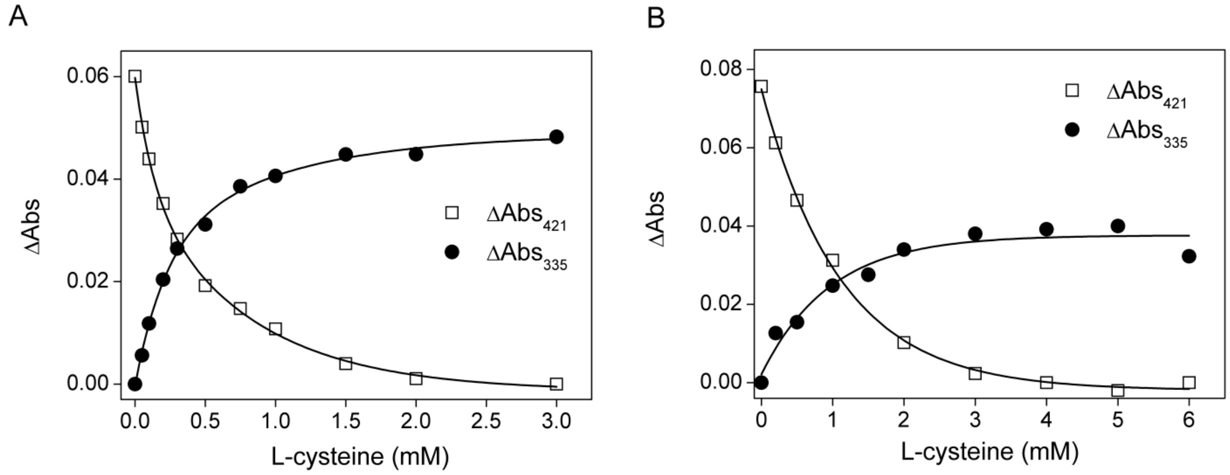

Figure S5. Spectral characterization of the interaction between TgCGL N360S and S77E variants and L-cysteine. Absorbance changes of N360S (**A**) and S77E (**B**) variants at 335 (solid circle) and 421 nm (open square) plotted against L-cysteine concentration. The continuous lines represent the theoretical curves according to Eqn. (3): 335 nm (K_{app} N360S = 335 ± 15 μM; K_{app} S77E = 677 ± 180 μM), and 421 nm (K_{app} N360S = 331 ± 20 μM; K_{app} S77E = 874 ± 87 μM).

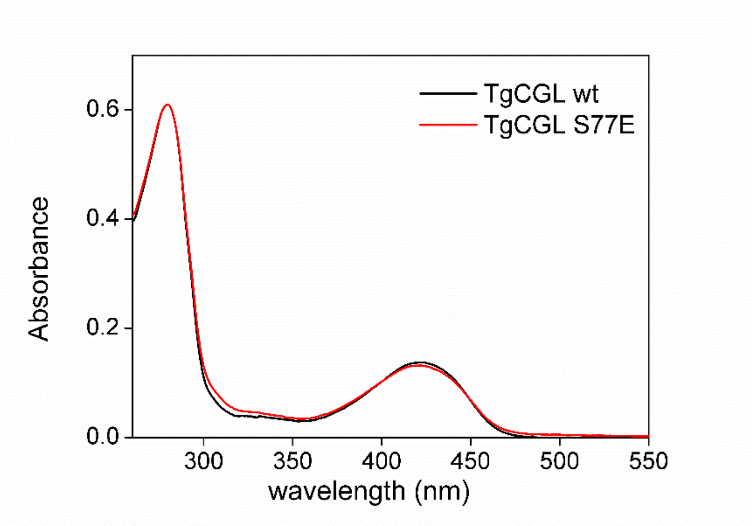

Figure S6. UV–visible absorption spectra of TgCGL wt (black line) and S77E variant (red line).

Table S1. List of mutagenic primers used to generate TgCGL active site variants. PF = Primer Forward, PR = Primer Reverse.

Mutation	Type of Primer	Primer Sequence
S77E	PF	GAGCAAAGGGTTCGAATATTCGCGAACTAGTAACCCG
	PR	CGGGTTACTAGTTCGCGAATATTCGAACCCTTTGCTC
S77A	PF	CTATTTGAGCAAAGGGTTCGCATATTCGCGAACTAGTAAC
	PR	GTTACTAGTTCGCGAATATGCGAACCCTTTGCTCAAATAG
N360S	PF	GGTCTTTGTGTGAAAGCCTGGGAGCGTGCG
	PR	CGCACGCTCCCAGGCTTTCACACAAAGACC
